# Tanned or Burned: The Role of Fire in Shaping Physical Seed Dormancy

**DOI:** 10.1371/journal.pone.0051523

**Published:** 2012-12-05

**Authors:** Bruno Moreira, Juli G. Pausas

**Affiliations:** Centro de Investigaciones sobre Desertificación (CIDE-CSIC), Montcada, Valencia, Spain; Institute of Botany, Czech Academy of Sciences, Czech Republic

## Abstract

Plant species with physical seed dormancy are common in mediterranean fire-prone ecosystems. Because fire breaks seed dormancy and enhances the recruitment of many species, this trait might be considered adaptive in fire-prone environments. However, to what extent the temperature thresholds that break physical seed dormancy have been shaped by fire (i.e., for post-fire recruitment) or by summer temperatures in the bare soil (i.e., for recruitment in fire-independent gaps) remains unknown. Our hypothesis is that the temperature thresholds that break physical seed dormancy have been shaped by fire and thus we predict higher dormancy lost in response to fire than in response to summer temperatures. We tested this hypothesis in six woody species with physical seed dormancy occurring in fire-prone areas across the Mediterranean Basin. Seeds from different populations of each species were subject to heat treatments simulating fire (i.e., a single high temperature peak of 100°C, 120°C or 150°C for 5 minutes) and heat treatments simulating summer (i.e., temperature fluctuations; 30 daily cycles of 3 hours at 31°C, 4 hours at 43°C, 3 hours at 33°C and 14 hours at 18°C). Fire treatments broke dormancy and stimulated germination in all populations of all species. In contrast, summer treatments had no effect over the seed dormancy for most species and only enhanced the germination in *Ulex parviflorus*, although less than the fire treatments. Our results suggest that in Mediterranean species with physical dormancy, the temperature thresholds necessary to trigger seed germination are better explained as a response to fire than as a response to summer temperatures. The high level of dormancy release by the heat produced by fire might enforce most recruitment to be capitalized into a single post-fire pulse when the most favorable conditions occur. This supports the important role of fire in shaping seed traits.

## Introduction

Plant species living in fire-prone ecosystems have several traits to cope with recurrent fires such as those related to resprouting and fire-stimulated recruitment. These two traits define the resprouter and seeder life histories, respectively [1]. Despite the fact that these traits increase plant fitness and thus are adaptive in fire-prone environments [2], to what extent they have been shaped by fire remains debatable [2], [3]. While resprouting enhances the persistence of individuals, fire-stimulated recruitment acts on a critical life stage for the persistence of the populations [1]. The consequence of fire-dependent germination is that the bulk of recruitment occurs post-fire and, because recurrent fires shorten the generation time of seeders (i.e., increase population turnover), they provide more opportunities for natural selection to act. Indeed, there is evidence that recurrent fires generate phenotypic [4] and genetic [5] differentiation in seeders. Thus, in fire-prone ecosystems, traits related to post-fire seedling recruitment are a likely target of strong selection.

One trait tightly related to post-fire seedling recruitment is seed dormancy, which allows species to maintain a persistent soil seed bank and delay germination until the conditions are optimal for seedling establishment. Seed dormancy is often broken by specific external stimuli that are coupled with those favorable conditions, allowing species to increase the probability of successful establishment. In mediterranean fire-prone ecosystems, the heat shock produced by wildfires has been shown to break seed dormancy and trigger germination in many species with physical dormancy (heat-stimulated germination, [6], [7]), particularly in Fabaceae and Cistaceae [8]–[11]. Seeds have specialized structures in the seedcoat (e.g., the strophiole in Fabaceae and the chalazal plug in Cistaceae) that move or become disrupted as a response to external factors, and thus allowing the water to surpass the impermeable seed coat layer(s) [12]. In some species, chemicals from the combustion might also enhance germination once physical dormancy has been broken by the heat shock (e.g. [13], [14]); however, in most species with impermeable seed coats, the embryo is nondormant [12] and breaking physical dormancy is enough for stimulating germination. Thus, physical seed dormancy is undoubtedly a trait that provides fitness benefits in fire-prone environments.

**Table 1:** List of species, number of populations studied per species (#Pop), location (Country) and date (month/year) of the seed collection, and seed age at the time of the experiment (in months).

Family	Species	# Pop	Country	Collection date	Seed age (months)
*Cistaceae*	*Fumana thymifolia*	2	Spain	07/2008	3
	*Cistus salviifolius*	6	Spain, Turkey	07/2009	1
	*Cistus albidus*	4	Spain	07/2008	3
	*Cistus parviflorus*	1	Turkey	07/2009	1
	*Cistus creticus*	3	Turkey	07/2009	1
*Fabaceae*	*Ulex parviflorus*	5	Spain	06/2008	4

Specific location of each population is given in Table S1.

Although, in mediterranean ecosystems, the regulation of dormancy release in species with physical seed dormancy is typically linked to fire, it could however be a response to the high summer temperatures in the bare soil (e.g., in vegetation gaps unrelated to fire). Because seeds in the seed bank are mostly located in the upper soil layer (e.g., seed density might be 2 to 5 times higher in the 0–2.5 cm layer than in the subsequent 2.5–5 cm layer [15]), they are exposed to temperature variations. Indeed, daily maximum summer temperatures registered in the bare soil can be relatively high (e.g., up to 50°C on the soil surface of fire breaks in eastern Spain [16]; up to 40–60°C in south-eastern Australia [17] ). Although temperatures registered during a fire might be much higher (e.g., in a spring burn, up to 150°C, 100°C and 50°C at 1 cm, 2.5 cm and 5 cm depth, respectively [18]), the cumulative effect throughout the summer could have a similar effect to a fire; i.e., the total heat dose might be similar and thus have analogous effects on seed dormancy. This has led to the suggestion that the temperature thresholds associated to the release from physical seed dormancy might have evolved independently of fire [3], [19], [20] and that the cumulative effect of soil temperatures during the summer might be more beneficial than the direct fire cues. This argument is based on the idea that cumulative summer temperatures should stimulate germination at the end of the summer (i.e., when conditions are less stressful) while seedling emergence resulting from the direct effect of fire could occur during the unfavorable conditions for recruitment of summer [21], [22]. However, even when dormancy has been broken, germination only occurs when favorable conditions are attained [12], [23], [24]. In addition, there is some evidence (e.g., comparing results by [22] and by [25]) that for similar maximum temperatures, a single heat dose (i.e., fire scenario) might be as much or even more efficient in breaking dormancy than the cumulative heat doses (i.e., summer scenario). This suggests that the release of physical dormancy might be determined by the maximum temperature experienced rather than by the amplitude of temperature fluctuations.

**Figure 1:**
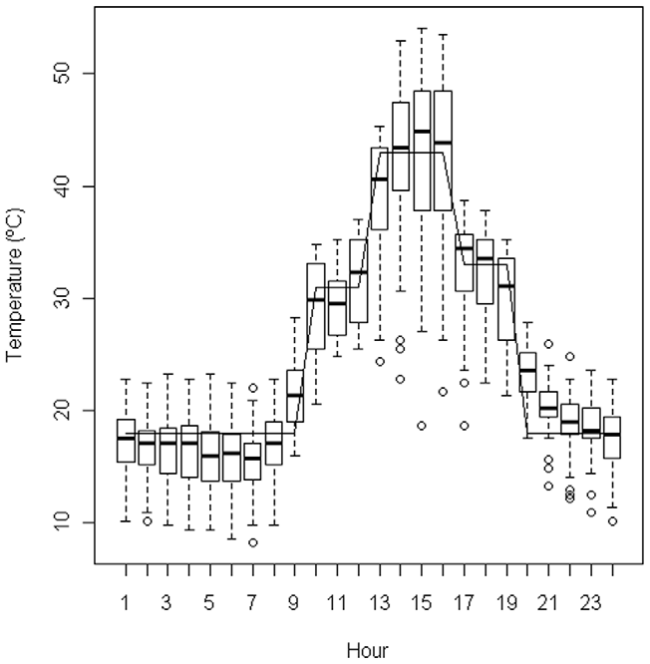
Hourly register of daily temperature (°C) observed during summer on the bare soil surface of a fire-break (with no slash). Data from a typically Mediterranean fire-prone area (August 1998, Valencia, eastern Spain [16]). Boxplots represent daily variability in temperature for each hour (n = 28 days). Dashed vertical lines represent the data within 1.5 interquartile range, and open circles are values outside this range (outliers). The continuous line represents the treatment applied to simulate summer temperatures.

The summer hypothesis for the physical seed dormancy proposes that it is an adaptation for recruiting in gaps, independently of the origin of the gap (i.e., seedling recruitment from the soil seedbank is not necessarily coupled with fire). One underlying premise of the summer hypothesis is the traditional belief that fire is a relatively new phenomenon (i.e., linked to human activities) and thus traits for post-fire persistence (such as physical seed dormancy) must be linked to another and earlier evolutionary pressure [3]. However, there is now a bulk of information suggesting that fire is an ancient phenomena in terrestrial ecosystems [26], [27] and that it has had a prominent role in shaping plant traits [2], [28]–[31]. In addition, there is no evidence that current summer conditions appeared earlier than fires in the evolutionary history and, in fact, predictable warm summers in most mediterranean ecosystems would also imply predictable fires (with the exception of central Chile where the Andes block summer storms reducing lighting and fire ignitions [23]). Thus, to what extent the temperature thresholds that break physical seed dormancy of species living in fire-prone mediterranean ecosystems have been shaped by fire or by summer temperatures remains unknown [2].

**Figure 2:**
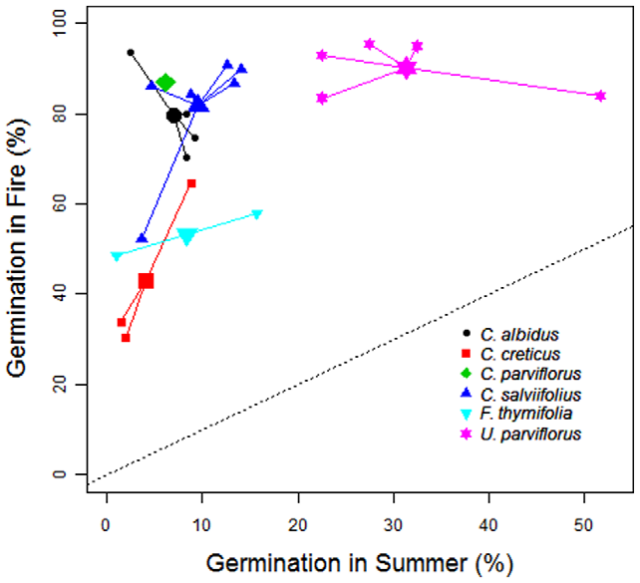
Germination (%) in the fire scenario and germination in the summer scenario. Relationship between the germination (%) after the treatment of 120°C for 5 minutes (Fire scenario, y-axis) and germination after the treatment simulating temperature fluctuations in the soil in open areas during August (Fig. 1; Summer scenario, x-axis). Intraspecific variability (i.e., among populations) is indicated by small symbols (mean population value) emerging from the large symbol (mean species value; F*umana thymifolia*, n = 2 populations; *Cistus salviifolius*, n = 6 populations; *Cistus albidus*, n = 4 populations; *Cistus parviflorus*, n = 1 population; *Cistus creticus*, n = 3 populations; *Ulex parviflorus*, n = 5 populations). The 1∶1 line is also shown (dotted line).

Previous research is ambiguous in disentangling the role of summer and fire in the regulation of dormancy release in species with physical seed dormancy due to the lack of an appropriate experimental approach [19]–[22]. Indeed, there is no single study simultaneously testing the two alternative hypotheses (fire *versus* summer) on species with physical seed dormancy. Our hypothesis is that fire shaped the temperature thresholds that break the physical seed dormancy in species from mediterranean fire-prone ecosystems. Thus, we predict that, in Mediterranean species with physical dormancy, fire produces a higher increase in the chances of recruitment (i.e., a higher increase in germination) than summer temperatures. To test this prediction we performed a germination experiment using different populations of six post-fire seeder species (Fabaceae and Cistaceae) distributed across the Mediterranean Basin. The experiment included treatments simulating both the temperatures registered in the soil during a fire and the temperature fluctuations observed in the bare soil during summer. Given that seed dormancy is heritable [32], [33], demonstrating that it provides higher chances of recruitment (i.e., higher potential fitness benefits) in response to fire than in response to summer temperatures would suggest this trait to be a good candidate for an adaptation to fire.

**Table 2:** Germination percentage (mean ± SE) of untreated seeds (Control), seeds treated with summer temperature fluctuations during 30 days (Summer 30) or 5 days (Summer 5) and heat-treated seeds (Fire treatments; 80°C, 100°C, 120°C or 150°C during 5 minutes), for each species and population (location of the different populations is given in Table S1).

				Summer treatments				Fire treatments							
Species	Pop.	Control		Summer 30		Summer 5		80°C		100°C		120°C		150°C	
*C. albidus*	P1	8±2.1	a	9±1.6	a	8±1.0	a	58±4.4	b	81±4.0	c	75±1.6	c	7±2.8	a
	P2	6±2.8	a	8±3.2	a	8±2.8	a	31±5.0	b	81±2.0	c	80±1.7	c	4±0.9	a
	P3	7±2.4	a	8±2.2	a	8±2.1	a	53±5.5	b	78±4.5	c	70±3.2	c	0±0	e
	P4	3±1.4	a	3±2.5	a	3±0.8	a	73±6.2	b	93±1.6	c	94±0.9	c	56±2.4	d
	Mean	6±1	a	7±2	a	6±1	a	54±9	b	83±3	c	80±5	c	17±13	a
*U. parviflorus*	P1	23±2.7	a	28±4.6	ab	41±4.6	bc	58±3.7	c	86±3.7	d	96±1.0	e	87±2.6	d
	P12	8±2.2	a	33±2.8	b	38±2.9	b	40±3.0	b	94±2.6	d	95±4.4	d	84±4	c
	P2	27±6.8	a	52±4.2	b	52±4.2	b	86±2.8	c	86±3.2	c	84±2.4	c	83±2.4	c
	P3	8±0.8	a	23±2.8	b	22±4.4	b	39±2.8	c	86±2.9	d	84±1.7	d	91±3.8	d
	P6	8±1.7	a	23±7.5	b	31±2.8	b	52±4.4	e	90±1.2	cd	93±1.9	c	84±2.6	d
	Mean	15±4	a	31±5	b	37±5	b	55±8	c	88±2	d	90±3	d	85±1	d
*C. creticus*	P13	4±0.8	a	2±1.0	a	-	-	-	-	12±9.3	b	34±5.2	c	73±15.3	d
	P14	10±1.9	a	9±1.1	a	-	-	-	-	51±16.3	b	65±3.9	c	95±3.3	d
	P16	0±0.0	a	2±1.4	a	-	-	-	-	2±1.2	a	30±7.0	b	70±5.2	c
	Mean	5±3	a	4±2	a	-	-	-	-	22±15	b	43±11	c	79±8	d
*C. parviflorus*	P16	4±2.3	a	6±2.1	a	-	-	-	-	94±6.0	b	87±6.9	b	99±0.6	c
*C. salviifolius*	P1	11±2.3	a	14±3.5	a	-	-	-	-	19±4.0	a	90±5.2	b	96±3.1	b
	P13	3±1.3	a	4±1.6	ab	-	-	-	-	10±4.4	b	52±7.7	c	98±1.0	d
	P14	7±1.3	a	5±1.1	a	-	-	-	-	92±7.0	b	86±7.9	b	92±3.7	b
	P16	10±2.5	a	13±2.6	a	-	-	-	-	32±19.0	b	91±2.1	c	99±0.6	d
	P5	3±1.1	a	9±2.0	a	-	-	-	-	28±17.8	b	84±2.4	c	97±1.8	d
	P8	4±1.8	a	13±2.0	a	-	-	-	-	56±16.7	b	87±5.3	c	100±0.0	d
	Mean	6±2	a	10±2	a	-	-	-	-	40±12	b	82±6	c	97±1	d
*F. thymifolia*	P5	8±1.2	a	16±2.2	a	-	-	-	-	35±3.2	b	58±2.7	c	83±3.1	d
	P6	1±0.6	a	1±0.6	a	-	-	-	-	36±8.0	b	49±7.2	b	78±3.5	c
	Mean	5±4	a	8±7	ab	-	-	-	-	35±0	b	53±5	c	80±2	d

Species means consider the variability between populations. For each species and population (Pop.), mean germination values of treatments with the same letter are not significantly different (P>0.05), after controlling for the false discovery rate.

## Methods

### Ethics statement

This work did not involve collecting animals or plants; only seeds were collected (from wild populations). None of species studies are endangered or protected, and all necessary permits for seed collection were obtained. Specifically, two of the sites are protected, and written permissions for seed collection were obtained from the corresponding authorities (“*Parque Natural del Carrascal de la Font Roja*” and “*Fundación Caja Castellón-Bancaja*”). For the remaining sites (non-protected), written permission for seed collection was not required, and the corresponding local authorities were properly notified.

### Species and seed collection

We selected six woody species with physical seed dormancy (i.e., water-impermeable seeds, Table 1) occurring in fire-prone areas of the Mediterranean Basin. All six species show evidence of post-fire seedling emergence [34]. We included one Fabaceae (*Ulex parviflorus* Pourr.), a family (and species) which had previously been shown to have germination stimulated by summer temperatures [16], [22] and five Cistaceae for which germination response to summer temperatures is unknown. The six species encompass the most abundant post-fire seeder species with physical seed dormancy in the Mediterranean Basin. Three species are abundant in the western part of Basin (*Fumana thymifolia* (L.) Spach, *Cistus albidus* L. and *U. parviflorus*) and seeds were collected in eastern Iberia (Spain), two are typical of the eastern part (*Cistus creticus* L. and *Cistus parviflorus* Lam.) and seeds were collected in south western Anatolia (Turkey) and one (*Cistus salviifolius* L.) occurs across all the basin and seeds were collected at both the eastern and the western sides (Turkey and Spain, [35]). For most species we sampled several populations, totaling 21 populations for the six species (Table 1 and Table S1). All seeds were collected from ripe fruits in wild populations during the dispersal period of each species (Table 1) from several individuals (>20 individuals for each species and population) spatially dispersed (>10 m from each other). Seeds were cleaned based on their density (i.e., excluding low density seeds that could be empty) using a seed cleaning equipment (vacuum aspirator). Seeds from the same species and population were pooled together for the germination experiments and were placed in aluminum pockets (ca. 50 apparently viable seeds per pocket; i.e., non predated seeds and with any perceptible damage). For each population and species, four aluminum pockets (four replicates) were randomly allocated to each treatment (see below).

### Fire and summer treatments

For each species, seeds from the different populations were subject to heat-treatments of high temperatures for a short period of time (i.e., fire treatments) and to treatments of temperature fluctuations at relatively low temperatures for a longer period of time (i.e., summer treatments). Treatments were applied to each replicate separately. The fire treatments consisted of submitting seeds to 100°C, 120°C and 150°C for 5 minutes, while in the summer treatments seeds were enclosed in a germination chamber for 30 days with daily cycles (24 hours) of 3 hours at 31°C, 4 hours at 43°C, 3 hours at 33°C and 14 hours at 18°C. These temperatures correspond to the temperature regime observed at the peak of summer on the soil surface of a Mediterranean environment (e.g., on the soil surface of a fire-break with no slash, Fig. 1; data from Ayora, Valencia, eastern Spain [16], which represents a typical environment where the studied species live). Indeed, this treatment is conservative in respect to our hypothesis as in natural conditions many seeds composing the soil seed bank might be buried and thus temperatures experienced by the seeds would be lower. For two of the species (*U. parviflorus* and *C. albidus*) we tested one additional fire treatment (80°C for 5 minutes) and one additional summer treatment in which seeds were enclosed in a germination chamber with daily cycles (24 hours) of 11 hours at 15°C and 13 hours at 45°C for 5 days. Although the 30 days treatment is more realistic (Fig. 1) we applied this 5-day treatment as it has been shown to successfully break dormancy of species with physical dormancy and thus could be used for comparison [16]. Fire treatments were performed using an electric oven in dark conditions at room moisture; summer treatments were performed in a germination chamber at dark conditions with 30% moisture.

After the respective treatments, seeds were set for germination in Petri dishes containing agar (0.9%) as substrate and incubated at 20°C in darkness. Seeds were put in dark conditions because these conditions are appropriate for the germination of many Mediterranean species [36]. Seed germination was monitored for 90 days, when no germination was recorded for 1 week. Seeds were scored as germinated and removed from the Petri dishes if radicle emergence exceeded 0.5–1 mm. At the end of the experiment, the initial number of seeds sown was corrected before statistical analysis by discarding the empty seeds (lacking embryo and storage tissue) detected during the experiment. The increment in the number of rotten seeds after treatment, in relation to control, was considered as seed mortality due to the treatment.

### Data analysis

We first tested, for each population of each species, whether final germination differed between treatments, using the analysis of deviance (GLM) with binomial error distribution. Then, for each species, we tested the effect of the treatments, accounting for the population variability, by using generalized linear mixed models (GLMM) with binomial error distribution, including treatment as fixed factor and population as random factor. Due to the large number of pairwise comparisons, we applied the false discovery rate correction [37] to control for the expected proportion of false discoveries amongst the rejected hypotheses; this is a less conservative criterion than the Bonferroni correction [38].

## Results

Fire treatments broke dormancy and stimulated germination in all species (Table 2 and Table S2). The summer treatments only enhanced germination of *U. parviflorus* (i.e., they had no significant effect over the germination of any of the Cistaceae species) and the magnitude of the stimulation by these treatments was lower than by the fire treatments (Table 2, Fig. 2 and Figure S1). These results were consistent across all populations (Table 2). The temperature threshold needed to break dormancy and maximize germination was species-dependent (Table 2); some species maintained a high germination level for a wide range of fire temperatures (e.g., *U. parviflorus* and *C. parviflorus*); others had their maximum germination at the highest heat doses (e.g., *C. creticus, C. salviifolius* and *F. thymifolia*); and still others showed seed mortality and consequently reduced germination at the highest heat dose applied (e.g., *C. albidus*). For the two species for which we tested a wider range of treatments (*U. parviflorus* and *C. albidus*), the heat treatment of 80°C for 5 minutes was insufficient to maximize germination (Table 2 and Table 3). In fact this treatment was the most variable among populations and for one of the populations of *U. parviflorus* this treatment was not significantly different from the summer treatments (P12 in Table 2). The two summer treatments were not significantly different for any of the species and populations (Table 2).

## Discussion

Our results suggest that for the species considered, which encompass the main post-fire obligate seeder species with physical dormancy from Mediterranean shrublands, dormancy release is higher in response to fire temperatures than to summer temperatures. That is, fire produces a higher increase in germination and consequently in the chance of recruitment than summer temperatures. These results were consistent for geographically distant populations and for all the studied species.

Many species with heat-stimulated germination have polymorphic seed pools [6]. Thus, while the bulk of the seed bank may respond to fire, a small proportion of the seeds may germinate in the absence of fire (e.g., in response to summer temperatures). Indeed, for some seeder species, especially for Fabaceae, summer temperatures also break physical dormancy of a significant proportion of the seeds (Table 2, [16], [21]). However, the magnitude of stimulated germination by summer temperatures is much lower than that resulting from the fire temperatures. Low levels of dormancy loss in response to summer temperatures might be important for the recruitment in fire-independent gaps or for the colonization of new areas, particularly in arid systems (e.g. *U. parviflorus* [39]; but also other species [12]). These species might display a gradual loss of dormancy over time to take advantage of occasional recruitment opportunities but maintain a considerable seed bank as a bet-hedging mechanism. This results in a large commitment of seeds to take advantage of the post-fire environment and a smaller commitment over multiple seasons. A similar pattern of response to summer temperatures may also be observed for other refractory mediterranean species without physical seed dormancy [17].

However, in fire-prone ecosystems, the spatial extent of fire-independent gaps is typically much reduced compared to the extent of gaps generated by fires (i.e., fire-independent gaps are smaller); in addition, in these ecosystems fire intervals are typically shorter than the lifespan of the dominant plants and thus fire-independent gaps (e.g., related to plant mortality) are also less frequent. Consequently, fire-independent recruitment of seeders should be less important and have less evolutionary implications than fire-dependent recruitment. Thus, it is not surprising that for many seeders the effectiveness of summer temperature fluctuations in stimulating germination is very limited (e.g., Cistaceae; Table 3, Fig. 2).

**Table 3:** Summary of the differences in germination between untreated seeds (Control), seeds subject to a summer treatment (Summer) and seeds subject to the fire treatments, for the six species studied.

Species	Germination
*Fumana thymifolia*	Control = Summer = Fire100<< Fire120<< Fire150
*Cistus salviifolius*	Control = Summer < Fire100<< Fire120<< Fire150
*Cistus albidus*	Control = Summer = Fire150< Fire80<< Fire100 = Fire120
*Cistus parviflorus*	Control = Summer << Fire100 = Fire120 = Fire150
*Cistus creticus*	Control = Summer << Fire100< Fire120<< Fire150
*Ulex parviflorus*	Control << Summer << Fire80<< Fire100 = Fire120 = Fire150

Fire treatment refers to the seeds submitted to 100°C, 120°C and 150°C for 5 minutes (Fire100, Fire120 and Fire150, respectively). Summer treatment refers to seeds exposed to temperature fluctuations for 30 days (Summer). For *C. albidus* and *U. parviflorus* an additional treatment of 80°C for 5 minutes (Fire80) is also included. The significance of the pairwise comparison between treatments is included ( = : not significant; <: P<0.05, <<: P<0.01). For full details of the statistical analysis see Table 2 and Table S2.

**Figure 3:**
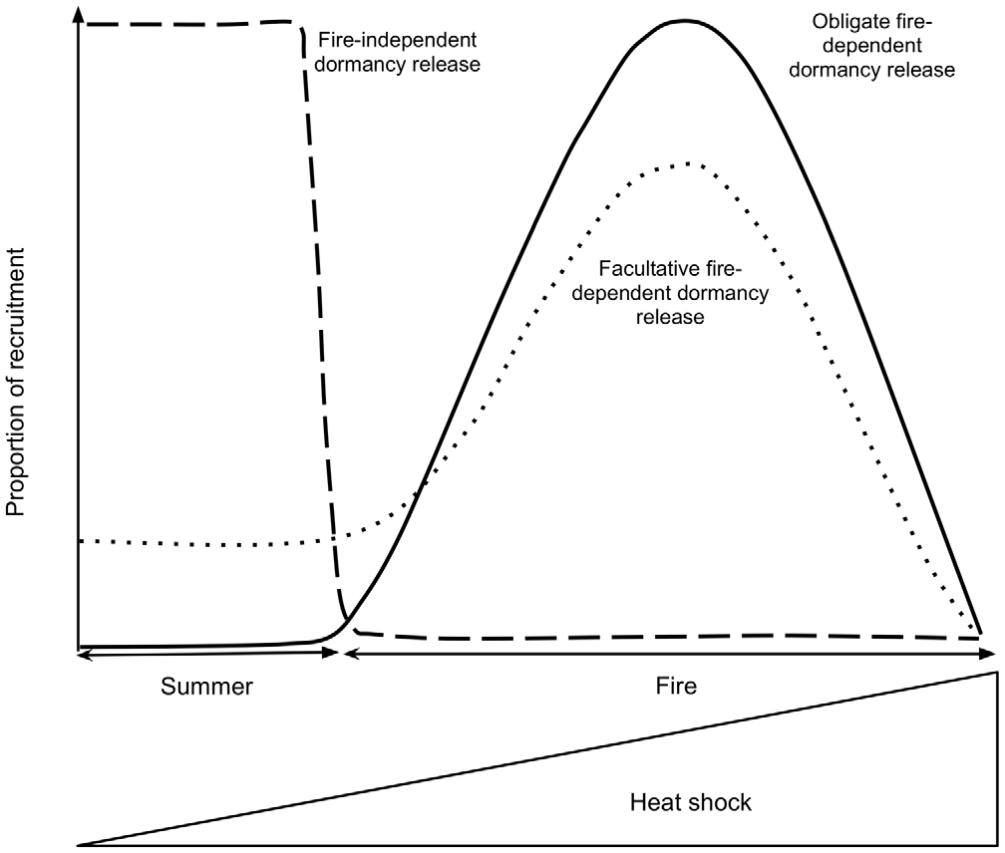
Conceptual model describing recruitment dynamics in mediterranean ecosystems for species with physical dormancy. The *x*-axis represents an increase in the heat doses reaching the soil (during summer or fires). The *y-*axis represents the proportion of recruitment associated with dormancy release, for species with different levels of fire-dependent recruitment. For some species, fire is not the main factor shaping seed dormancy and thus they have fire-independent dormancy release and recruitment (dashed line). However, species living in fire prone mediterranean ecosystems have mostly fire-dependent dormancy release, with recruitment strictly related to fire (obligate fire-dependent dormancy release, continuous line) or with a small proportion of recruitment independent of fire (facultative fire-dependent dormancy release, dotted line).

Our results might explain the observed dynamics of seedling establishment in mediterranean shrublands where there is a flush of germination after fire with very little (if any) effective recruitment between fires [7]. The emergence of species with fire-dependent dormancy release (e.g., *Cistus*) occurs in the post-fire environment, while in species with a significant proportion of seeds with fire-independent dormancy release (e.g., *Ulex parviflorus*) some emergence is also observed in fire-independent gaps ([39], [40], Fig. 3). Indeed, for different species of *Cistus*, successful establishment is restricted to the immediate post-fire period [40], [41].

The lower effect of summer temperature fluctuations suggests that cumulative periods of relatively low temperatures (i.e., summer heat dose) are less effective in breaking physical seed dormancy than a single high temperature peak (i.e., fire heat dose). This agrees with the importance of fire intensity at the soil level in fire-prone ecosystems [42]. Fire intensity influences seedling recruitment [43], [44], [45] because seeds have different heat tolerance [46] and different temperature thresholds needed for dormancy release (Tables 2 and Table 3; [44], [47]). For instance, obligate (non-resprouting) seeders have higher heat tolerance, heat-stimulated germination and post-fire seedling emergence than facultative (resprouting) seeders and this is particularly evident for high intensity fires (i.e., high heat doses, [48], [49]. In addition, in Mediterranean shrublands, there is evidence that obligate seeders have evolved flammability-enhancing traits [4], [50] that increase soil temperatures during fires, ensuring that seed dormancy is broken and germination is triggered by fire. Fire intensity also negatively affects the resprouting capacity [51], [52], [53] and thus by increasing flammability and the temperature threshold for breaking seed dormancy, seeder species might also be favored in relation to resprouters.

In frequently disturbed mediterranean ecosystems, despite the fact that heat from fire is the main factor driving dormancy release, summer temperatures might also have an important role in shaping physical seed dormancy. That is, physical dormancy might have evolved in such a way that the temperature threshold associated with the dormancy release is above the maximum summer temperatures. Thus, seeds from plants growing in hotter conditions might have dormancy-breaking thresholds higher than those growing in cooler conditions (i.e., dormancy-breaking thresholds might depend of the local climatic conditions). This ensures the maintenance of a persistent seed bank until a fire occurs [54].

Our conclusions are based on the range of summer temperatures tested, which come from field observations in the soil surface (Fig. 1). In fact, using these temperatures is a conservative approach because in natural conditions many seeds in the soil seed bank are buried and thus they experience lower summer temperatures. We cannot discard that extreme heat waves experienced during summer periods can increase soil temperatures above the levels tested; however, we expect that this extreme effects to be rare. The increased frequency of heat waves, due to global warming, might increase dormancy loss, especially in populations growing under cooler conditions [54].

Seed dormancy and germination are key traits in plant evolution, not only because they determine the persistence of populations but also because of the strong selective pressure exerted by the conditions that plants experience for germination. Our results suggest that physical dormancy in mediterranean ecosystems has evolved in the presence of frequent fires in such a way that germination in seeders is mainly capitalized to a single post-fire pulse. That is, the heat from fire produces high levels of dormancy release that might be responsible for the single post-fire germination pulse occurring just after the first post-fire rains. This semelparity-like strategy [55] allows maximizing germination at the most favorable moment for recruitment (e.g., lower competition and higher resources availability).

The link between fire and dormancy-breaking can also be observed at a biogeographical scale. For instance, the rare fire-dependent recruitment observed in central Chile [7], [29], might be explained by the fact that fires are historically rare, compared with the other Mediterranean climate regions where both fires and fire-dependent recruitment are very common [1], [29]. In fact, fire-stimulated germination in Chile is mainly observed in alien species and in native annuals, because annuals had time to adapt to anthropogenic fires [56], [57].

Despite the fact that some species with physical seed dormancy in fire-prone ecosystems may have a fraction of seeds with fire-independent germination, our results support the hypothesis that temperature thresholds that break physical seed dormancy are better explained as a response to fire than as a response to the summer temperatures. This together with other recent studies (e.g., [30], [56]), highlights the role of fire in driving the evolution of seed traits in mediterranean ecosystems.

## Supporting Information

Figure S1 **Germination (%) in control conditions (untreated seeds), in the fire scenario and in the summer scenario.** Relationship of the germination (%) of untreated seeds (Control) with the germination after the treatment of 120°C for 5 minutes (Fire; filled symbols) and after the treatment simulating temperature fluctuations in the soil (Summer; open symbols). Intraspecific variability (i.e., among populations) is indicated by small symbols (mean population value) emerging from the large symbol (mean species value; *Fumana thymifolia*, n = 2 populations; *Cistus salviifolius*, n = 6 populations; *Cistus albidus*, n = 4 populations; *Cistus parviflorus*, n = 1 population; *Cistus creticus*, n = 3 populations; *Ulex parviflorus*, n = 5 populations). The 1∶1 line is also shown (dotted line).
(TIFF)

Table S1 **Location and country (TR: south west Turkey; ES: eastern Spain) of the populations used in the study for each species (FTH:** ***Fumana thymifolia*** **; CSA:** ***Cistus salviifolius*** **; CAL:** ***Cistus albidus*** **; CPA:** ***Cistus parviflorus*** **; CCR:** ***Cistus creticus*** **; UPA:** ***Ulex parviflorus*** **).**
(DOC)

Table S2 **Statistical analyses (GLMM) of pairwise differences in germination between treatments (Control, Summer and Fire) for each of the six species studied.** The significance of the treatment was tested including population as a random factor (ns refers to p>0.05); p-values are those obtained after the false discovery rate correction. For each species, parameter estimates (and S.E.) refer to treatment B in relation to A; that is, positive estimated values indicate higher germination in treatment B while negative estimated values indicate higher germination in treatment A. Control refers to untreated seeds (Control), fire treatment refers to the seeds submitted to 80°C, 100°C, 120°C and 150°C for 5 minutes (Fire 80, Fire 100, Fire 120 and Fire 150, respectively); and the summer treatments refer to seeds exposed to temperature fluctuations for 5 or 30 days (Summer 5 and Summer 30, respectively). A summary of these results is provided in Table 2 (Species mean) and Table 3 of the main text.
(DOC)

## References

[1] Pausas JG, Keeley JE, Keith DA, Bradstock RA (2004) Plant functional traits in relation to fire in crown-fire ecosystems. Ecology 85: 1085–1100.

[2] Keeley JE, Pausas JG, Rundel PW, Bond WJ, Bradstock RA (2011) Fire as an evolutionary pressure shaping plant traits. Trends in Plant Science 16: 406–411. 21571573 10.1016/j.tplants.2011.04.002

[3] Bradshaw SD, Dixon KW, Hopper SD, Lambers H, Turner SR (2011) Little evidence for fire-adapted plant traits in mediterranean climate regions. Trends in Plant Science 16: 69–76. 21095155 10.1016/j.tplants.2010.10.007

[4] Pausas JG, Alessio GA, Moreira B, Corcobado G (2012) Fires enhance flammability in *Ulex parviflorus* . New Phytologist 193: 18–23. 22039968 10.1111/j.1469-8137.2011.03945.x

[5] Segarra-Moragues JG, Ojeda F (2010) Postfire response and genetic diversity in *Erica coccinea*: connecting population dynamics and diversification in a biodiversity hotspot. Evolution 64: 3511–3524. 20561051 10.1111/j.1558-5646.2010.01064.x

[6] Keeley JE (1991) Seed germination and life history syndromes in the California chaparral. The Botanical Review 57: 81–116.

[7] Keeley JE (1995) Seed germination patterns in fire-prone mediterranean-climate regions. In: Arroyo MTK, Zedler PH, Fox MD, editors. Ecology and biogeography of mediterranean ecosystems in Chile, California and Australia. San Diego: Academic Press. pp. 239–273.

[8] Bell D, Plummer J, Taylor S (1993) Seed germination ecology in southwestern Western Australia. The Botanical Review 59: 24–73.

[9] Herranz JM, Ferrandis P, Martínez-Sánchez JJ (1998) Influence of heat on seed germination of seven Mediterranean Leguminosae species. Plant Ecology 136: 95–103.

[10] Herranz JM, Ferrandis P, Martinez-Sánchez JJ (1999) Influence of heat on seed germination of nine woody Cistaceae species. International Journal of Wildland Fire 9: 173–182.

[11] Moreira B, Tavsanoglu Ç, Pausas J (2011) Local versus regional intraspecific variability in regeneration traits. Oecologia 168: 671–677. 21935664 10.1007/s00442-011-2127-5

[12] Baskin C, Baskin J (1998) Seeds, ecology, biogeography, and evolution of dormancy and germination. San Diego: Academic Press.

[13] Keith DA (1997) Combined effects of heat shock, smoke and darkness on germination of *Epacris stuartii* Stapf., an endangered fire-prone Australian shrub. Oecologia 112: 340–344. 28307482 10.1007/s004420050318

[14] Thomas PB, Morris EC, Auld TD (2003) Interactive effects of heat shock and smoke on germination of nine species forming soil seed banks within the Sydney region. Austral Ecology 28: 674–683.

[15] Clemente A, Rego F, Correia O (2007) Seed bank dynamics of two obligate seeders, *Cistus monspeliensis* and *Rosmarinus officinalis* , in relation to time since fire. Plant Ecology 190: 175–188.

[16] Baeza MJ, Roy J (2008) Germination of an obligate seeder (*Ulex parviflorus*) and consequences for wildfire management. Forest Ecology and Management 256: 685–693.

[17] Tieu A, Dixon KW, Meney KA, Sivasithamparam K (2001) The interaction of heat and smoke in the release of seed dormancy in seven species from southwestern Western Australia. Annals of Botany 88: 259–265.

[18] Trabaud L (1979) Etude du comportement du feu dans la garrigue de chêne kermes à partir des températures et des vitesses de propagation. Annales des Sciences Forestieres 36: 13–38.

[19] Buhk C, Hensen I (2006) ''Fire seeders'' during early post-fire succession and their quantitative importance in south-eastern Spain. Journal of Arid Environments 66: 193–209.

[20] Luna B, Moreno JM, Cruz A, Fernández-González F (2007) Heat-shock and seed germination of a group of Mediterranean plant species growing in a burned area: An approach based on plant functional types. Environmental and Experimental Botany 60: 324–333.

[21] Auld TD, Bradstock RA (1996) Soil temperatures after the passage of a fire: do they influence the germination of buried seeds? Australian Journal of Ecology 21: 106–109.

[22] Santana VM, Bradstock RA, Ooi MKJ, Denham AJ, Auld TD, et al (2010) Effects of soil temperature regimes after fire on seed dormancy and germination in six Australian Fabaceae species. Australian Journal of Botany 58: 539–545.

[23] Fenner M, Thompson K (2005) The ecology of seeds: Cambridge University Press.

[24] Thompson K, Ooi MKJ (2010) To germinate or not to germinate: more than just a question of dormancy. Seed Science Research 20: 209–211.

[25] Morrison DA, Mcclay K, Porter C, Rish S (1998) The role of the lens in controlling heat-induced breakdown of testa-imposed dormancy in native Australian legumes. Annals of Botany 82: 35–40.

[26] Pausas JG, Keeley JE (2009) A burning story: the role of fire in the history of life. BioScience 59: 593–601.

[27] Bond WJ, Scott AC (2010) Fire and the spread of flowering plants in the Cretaceous. New Phytologist 188: 1137–1150. 20819174 10.1111/j.1469-8137.2010.03418.x

[28] He T, Lamont BB, Downes KS (2011) Banksia born to burn. New Phytologist 191: 184–196. 21388378 10.1111/j.1469-8137.2011.03663.x

[29] Keeley JE, Bond WJ, Bradstock RA, Pausas JG, Rundel PW (2012) Fire in mediterranean ecosystems: ecology, evolution and management: Cambridge University Press.

[30] Pausas JG, Schwilk DW (2012) Fire and plant evolution. New Phytologist 193: 301–303. 22221150 10.1111/j.1469-8137.2011.04010.x

[31] He T, Pausas JG, Belcher CM, Schwilk DW, Lamont BB (2012) Fire-adapted traits of Pinus arose in the fiery Cretaceous. New Phytologist 194: 751–759. 22348443 10.1111/j.1469-8137.2012.04079.x

[32] Baskin JM, Baskin CC, Li X (2000) Taxonomy, anatomy and evolution of physical dormancy in seeds. Plant Species Biology 15: 139–152.

[33] Huang X, Schmitt J, Dorn L, Griffith C, Effgen S, et al (2010) The earliest stages of adaptation in an experimental plant population: strong selection on QTLS for seed dormancy. Molecular Ecology 19: 1335–1351. 20149097 10.1111/j.1365-294X.2010.04557.x

[34] Paula S, Arianoutsou M, Kazanis D, Tavsanoglu Ç, Lloret F, et al (2009) Fire-related traits for plant species of the Mediterranean Basin. Ecology 90: 1420–1420.

[35] Moreira B, Tormo J, Estrelles E, Pausas JG (2010) Disentangling the role of heat and smoke as germination cues in Mediterranean Basin flora. Annals of Botany 105: 627–635. 20181568 10.1093/aob/mcq017 PMC2850801

[36] Thanos CA, Georghiou K, Douma DJ, Marangaki CJ (1991) Photoinhibition of seed germination in Mediterranean maritime plants. Annals of Botany 68: 469–475.

[37] Benjamini Y, Yekutieli D (2001) The control of the false discovery rate in multiple testing under dependency. Annals of Statistics 29: 1165–1188.

[38] Moran MD (2003) Arguments for rejecting the sequential Bonferroni in ecological studies. Oikos 100: 403–405.

[39] Baeza MJ, Santana VM, Pausas JG, Vallejo VR (2011) Successional trends in standing dead biomass in Mediterranean basin species. Journal of Vegetation Science 22: 467–474.

[40] Santana VM, Baeza MJ, Maestre FT (2012) Seedling establishment along post-fire succession in Mediterranean shrublands dominated by obligate seeders. Acta Oecologica 39: 51–60.

[41] Moreno JM, Zuazua E, Pérez B, Luna B, Velasco A, et al (2011) Rainfall patterns after fire differentially affect the recruitment of three Mediterranean shrubs. Biogeosciences Discuss 8: 5761–5786.

[42] Pausas JG, Moreira B (2012) Flammability as a biological concept. New Phytologist 194: 610–613. 22489901 10.1111/j.1469-8137.2012.04132.x

[43] Bond W, Roux D, Erntzen R (1990) Fire intensity and regeneration of myrmecochorous Proteaceae. South African Journal of Botany 56: 326–330.

[44] Thanos CA, Georghiou K, Kadis C, Pantazi C (1992) Cistaceae: a plant family with hard seeds. Israel Journal of Botany 41: 251–263.

[45] Schwilk DW (2003) Flammability is a niche construction trait: canopy architecture affects fire intensity. The American Naturalist 162: 725–733. 10.1086/379351 14737710

[46] Bell DT, Williams DS (1998) Tolerance of thermal shock in seeds. Australian Journal of Botany 46: 221–233.

[47] Auld TD, O'Connell MA (1991) Predicting patterns of post-fire germination in 35 eastern Australian Fabaceae. Australian Journal of Ecology 16: 53–70.

[48] Moreno JM, Oechel WC (1991) Fire intensity effects on germination of shrubs and herbs in Southern California Chaparral. Ecology 72: 1993–2004.

[49] Paula S, Pausas JG (2008) Burning seeds: germinative response to heat treatments in relation to resprouting ability. Journal of Ecology 96: 543–552.

[50] Saura-Mas S, Paula S, Pausas J, Lloret F (2010) Fuel loading and flammability in the Mediterranean Basin woody species with different post-fire regenerative strategies. International Journal of Wildland Fire 19: 783–794.

[51] Moreno JM, Oechel WC (1991) Fire intensity and herbivory effects on post-fire resprouting of *Adenostoma fasciculatum* in southern California chaparral. Oecologia 85: 429–433. 28312050 10.1007/BF00320621

[52] Lloret F, Lopez-Soria L (1993) Resprouting of *Erica multiflora* after experimental fire treatments. Journal of Vegetation Science 4: 367–374.

[53] Vesk PA, Warton DI, Westoby M (2004) Sprouting by semi-arid plants: testing a dichotomy and predictive traits. Oikos 107: 72–89.

[54] Ooi M, Auld T, Denham A (2012) Projected soil temperature increase and seed dormancy response along an altitudinal gradient: implications for seed bank persistence under climate change. Plant and Soil 353: 289–303.

[55] Keeley JE (1986) Resilience of mediterranean shrub communities to fire. In: Dell B, Hopkins AJM, Lamont BB, editors. Resilience in mediterranean-type ecosystems. The Netherlands: Dr. W. Junk, Dordrecht. pp. 95–112.

[56] Gómez-González S, Torres-Díaz C, Bustos-Schindler C, Gianoli E (2011) Anthropogenic fire drives the evolution of seed traits. Proceedings of the National Academy of Sciences 108: 18743–18747. 10.1073/pnas.1108863108 PMC3219139 22065739

[57] Gómez-González S, Torres-Díaz C, Valencia G, Torres-Morales P, Cavieres LA, et al (2011) Anthropogenic fires increase alien and native annual species in the Chilean coastal matorral. Diversity and Distributions 17: 58–67.

