# Supplementary figures and images for: Tanned or Burned: The Role of Fire in Shaping Physical Seed Dormancy
Source: PLoS One. 2012 Dec 5;7(12):e51523. doi: 10.1371/journal.pone.0051523 (PMC3515543; doi:10.1371/journal.pone.0051523)

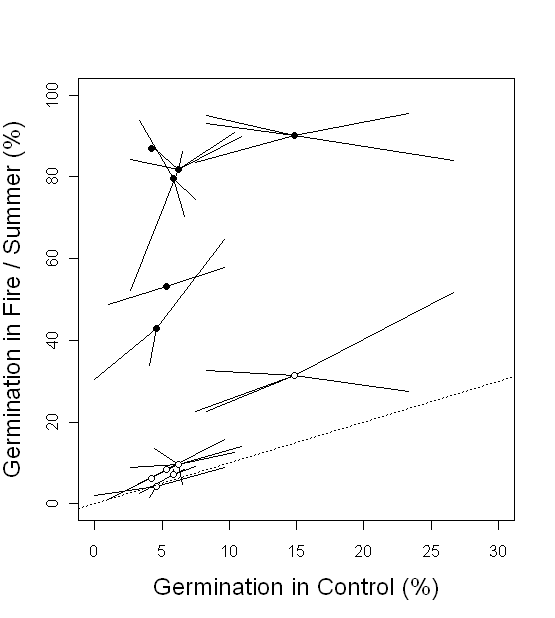

Supplement: Figure S1 — Germination (%) in control conditions (untreated seeds), in the fire scenario and in the summer scenario. Relationship of the germination (%) of untreated seeds (Control) with the germination after the treatment of 120°C for 5 minutes (Fire; filled symbols) and after the treatment simulating temperature fluctuations in the soil (Summer; open symbols). Intraspecific variability (i.e., among populations) is indicated by small symbols (mean population value) emerging from the large symbol (mean species value; Fumana thymifolia, n = 2 populations; Cistus salviifolius, n = 6 populations; Cistus albidus, n = 4 populations; Cistus parviflorus, n = 1 population; Cistus creticus, n = 3 populations; Ulex parviflorus, n = 5 populations). The 1∶1 line is also shown (dotted line). (TIFF) [file pone.0051523.s001.tiff]
