# Supplementary material for: Tanned or Burned: The Role of Fire in Shaping Physical Seed Dormancy
Source: PLoS One. 2012 Dec 5;7(12):e51523. doi: 10.1371/journal.pone.0051523 (PMC3515543; doi:10.1371/journal.pone.0051523)
Supplement: Table S1 — Location and country (TR: south west Turkey; ES: eastern Spain) of the populations used in the study for each species (FTH: Fumana thymifolia ; CSA: Cistus salviifolius ; CAL: Cistus albidus ; CPA: Cistus parviflorus ; CCR: Cistus creticus ; UPA: Ulex parviflorus ). (DOC) [file pone.0051523.s002.doc]

**Table S1.** Location and country (TR: south west Turkey; ES: eastern Spain) of the populations used in the study for each species (FTH: *Fumana thymifolia*; CSA: *Cistus salviifolius*; CAL: *Cistus albidus*; CPA: *Cistus parviflorus*; CCR: *Cistus creticus*; UPA: *Ulex parviflorus*).

| **Code** | **Location (country)** | **Coordinates (lat, long)** | **Species** |
| --- | --- | --- | --- |
| P1 | Serra Espadà (ES) | 39.874834, -0.372591 | CAL; CSA; UPA |
| P2 | Font Roja (ES) | 38.668892, -0.519791 | CAL; UPA |
| P3 | Dos Aguas (ES) | 39.300432, -0.788097 | CAL; UPA |
| P4 | Chiva (ES) | 39.530658, -0.809555 | CAL |
| P5 | Serra Calderona (ES) | 39.753657, -0.494385 | CSA; FTH |
| P6 | Ayora (ES) | 39.116667, -0.950000 | UPA; FTH |
| P8 | Desert de les Palmes (ES) | 40.067325, 0.040802 | CSA |
| P12 | Barranc dels Horts (ES) | 40.401536, -0.086174 | UPA |
| P13 | Marmaris (TR) | 36.914640, 28.242170 | CCR; CSA |
| P14 | Fethiye (TR) | 36.725866, 28.990324 | CCR; CSA |
| P16 | Datça (TR) | 36.681477, 27.404902 | CCR; CPA; CSA |
