# Supplementary material for: Tanned or Burned: The Role of Fire in Shaping Physical Seed Dormancy
Source: PLoS One. 2012 Dec 5;7(12):e51523. doi: 10.1371/journal.pone.0051523 (PMC3515543; doi:10.1371/journal.pone.0051523)
Supplement: Table S2 — Statistical analyses (GLMM) of pairwise differences in germination between treatments (Control, Summer and Fire) for each of the six species studied. The significance of the treatment was tested including population as a random factor (ns refers to p>0.05); p-values are those obtained after the false discovery rate correction. For each species, parameter estimates (and S.E.) refer to treatment B in relation to A; that is, positive estimated values indicate higher germination in treatment B while negative estimated values indicate higher germination in treatment A. Control refers to untreated seeds (Control), fire treatment refers to the seeds submitted to 80°C, 100°C, 120°C and 150°C for 5 minutes (Fire 80, Fire 100, Fire 120 and Fire 150, respectively); and the summer treatments refer to seeds exposed to temperature fluctuations for 5 or 30 days (Summer 5 and Summer 30, respectively). A summary of these results is provided in Table 2 (Species mean) and Table 3 of the main text. (DOC) [file pone.0051523.s003.doc]

**Table S2**. Statistical analyses (GLMM) of pairwise differences in germination between treatments (Control, Summer and Fire) for each of the six species studied. The significance of the treatment was tested including population as a random factor (ns refers to p > 0.05); p-values are those obtained after the false discovery rate correction. For each species, parameter estimates (and S.E.) refer to treatment B in relation to A; that is, positive estimated values indicate higher germination in treatment B while negative estimated values indicate higher germination in treatment A. Control refers to untreated seeds (Control), fire treatment refers to the seeds submitted to 80ºC, 100ºC, 120ºC and 150ºC for 5 minutes (Fire 80, Fire 100, Fire 120 and Fire 150, respectively); and the summer treatments refer to seeds exposed to temperature fluctuations for 5 or 30 days (Summer 5 and Summer 30, respectively). A summary of these results is provided in Table 2 (Species mean) and Table 3 of the main text.

| Species | A | B | Estimate | S.E. | z value | Pr(>|z|) |
| --- | --- | --- | --- | --- | --- | --- |
| *C. albidus* | Control | Summer 5 | 0.0959 | 0.2724 | 0.35 | ns |
|  |  | Summer 30 | 0.1868 | 0.2698 | 0.69 | ns |
|  |  | Fire 80 | 2.9414 | 0.3795 | 7.75 | < 0.00001 |
|  |  | Fire 100 | 4.4531 | 0.3095 | 14.39 | < 0.00001 |
|  |  | Fire 120 | 4.2650 | 0.3980 | 10.71 | < 0.00001 |
|  |  | Fire 150 | -0.2955 | 1.3295 | -0.22 | ns |
|  |  |  |  |  |  |  |
|  | Summer 5 | Summer 30 | -0.0930 | 0.2587 | -0.36 | ns |
|  |  | Fire 80 | 2.8330 | 0.3749 | 7.56 | < 0.00001 |
|  |  | Fire 100 | 4.3448 | 0.3039 | 14.29 | < 0.00001 |
|  |  | Fire 120 | 4.1565 | 0.3937 | 10.56 | < 0.00001 |
|  |  | Fire 150 | -0.4039 | 1.3282 | -0.304 | ns |
|  |  |  |  |  |  |  |
|  | Summer 30 | Fire 80 | 2.7339 | 0.3711 | 7.37 | < 0.00001 |
|  |  | Fire 100 | 4.2457 | 0.2992 | 14.19 | < 0.00001 |
|  |  | Fire 120 | 4.0574 | 0.3901 | 10.40 | < 0.00001 |
|  |  | Fire 150 | -0.2677 | 1.3077 | -0.20 | ns |
|  |  |  |  |  |  |  |
|  | Fire 80 | Fire 100 | 1.5095 | 0.2435 | 6.20 | < 0.00001 |
|  |  | Fire 120 | 1.3232 | 0.3145 | 4.21 | < 0.001 |
|  |  | Fire 150 | -3.0914 | 1.0530 | -2.94 | 0.02 |
|  |  |  |  |  |  |  |
|  | Fire 100 | Fire 120 | -0.1906 | 0.1662 | -1.15 | ns |
|  |  | Fire 150 | -4.3494 | 0.7838 | -5.55 | < 0.00001 |
|  |  |  |  |  |  |  |
|  | Fire 120 | Fire 150 | -4.2124 | 0.7391 | -5.70 | < 0.00001 |
|  |  |  |  |  |  |  |
|  |  |  |  |  |  |  |
| *C. creticus* | Control | Summer 30 | 0.2329 | 0.5647 | 0.41 | ns |
|  |  | Fire 100 | 1.6080 | 0.3591 | 4.48 | < 0.0001 |
|  |  | Fire 120 | 3.3047 | 0.5504 | 6.00 | < 0.00001 |
|  |  | Fire 150 | 5.2059 | 0.6044 | 8.61 | < 0.00001 |
|  |  |  |  |  |  |  |
|  |  |  |  |  |  |  |
|  | Summer 30 | Fire 100 | 1.5438 | 0.4794 | 3.22 | < 0.01 |
|  |  | Fire 120 | 3.1357 | 0.2520 | 12.44 | < 0.00001 |
|  |  | Fire 150 | 4.9889 | 0.2981 | 16.73 | < 0.00001 |
|  |  |  |  |  |  |  |
|  | Fire 100 | Fire 120 | 1.5439 | 0.5179 | 2.98 | 0.02 |
|  |  | Fire 150 | 3.4190 | 0.4611 | 7.41 | < 0.00001 |
|  |  |  |  |  |  |  |
|  | Fire 120 | Fire 150 | 1.8604 | 0.2379 | 7.82 | < 0.00001 |
|  |  |  |  |  |  |  |
|  |  |  |  |  |  |  |
| *C. parviflorus* | Control | Summer 30 | 0.4216 | 0.4681 | 0.90 | ns |
|  |  | Fire 100 | 5.8597 | 0.4768 | 12.29 | < 0.00001 |
|  |  | Fire 120 | 5.0531 | 0.4246 | 11.90 | < 0.00001 |
|  |  | Fire 150 | 8.2810 | 1.0660 | 7.77 | < 0.00001 |
|  |  |  |  |  |  |  |
|  |  |  |  |  |  |  |
|  | Summer 30 | Fire 100 | 5.4380 | 0.4310 | 12.62 | < 0.00001 |
|  |  | Fire 120 | 4.6315 | 0.3725 | 12.43 | < 0.00001 |
|  |  | Fire 150 | 7.8599 | 1.0463 | 7.51 | < 0.00001 |
|  |  |  |  |  |  |  |
|  | Fire 100 | Fire 120 | -0.8066 | 0.3833 | -2.10 | ns |
|  |  | Fire 150 | 2.4218 | 1.0501 | 2.31 | ns |
|  |  |  |  |  |  |  |
|  | Fire 120 | Fire 150 | 3.2284 | 1.0275 | 3.14 | 0.01 |
|  |  |  |  |  |  |  |
|  |  |  |  |  |  |  |
| *C. salviifolius* | Control | Summer 30 | 0.4483 | 0.1780 | 2.52 | ns |
|  |  | Fire 100 | 2.3844 | 0.6348 | 3.76 | < 0.01 |
|  |  | Fire 120 | 4.4691 | 0.1871 | 23.89 | < 0.00001 |
|  |  | Fire 150 | 6.5889 | 0.5106 | 12.90 | < 0.00001 |
|  |  |  |  |  |  |  |
|  |  |  |  |  |  |  |
|  | Summer 30 | Fire 100 | 1.9239 | 0.6956 | 2.77 | 0.04 |
|  |  | Fire 120 | 4.0255 | 0.1856 | 21.69 | < 0.00001 |
|  |  | Fire 150 | 6.1424 | 0.3811 | 16.12 | < 0.00001 |
|  |  |  |  |  |  |  |
|  | Fire 100 | Fire 120 | 2.0996 | 0.5630 | 3.73 | < 0.01 |
|  |  | Fire 150 | 4.1205 | 0.8524 | 4.83 | < 0.0001 |
|  |  |  |  |  |  |  |
|  | Fire 120 | Fire 150 | 2.1017 | 0.5306 | 3.96 | < 0.001 |
|  |  |  |  |  |  |  |
|  |  |  |  |  |  |  |
| *F. thymifolia* | Control | Summer 30 | 0.3106 | 0.3699 | 0.84 | ns |
|  |  | Fire 100 | 2.7515 | 0.8592 | 3.20 | < 0.01 |
|  |  | Fire 120 | 3.4855 | 0.7390 | 4.72 | < 0.0001 |
|  |  | Fire 150 | 4.7452 | 0.7661 | 6.19 | < 0.00001 |
|  |  |  |  |  |  |  |
|  |  |  |  |  |  |  |
|  | Summer 30 | Fire 100 | 2.4790 | 1.0740 | 2.31 | ns |
|  |  | Fire 120 | 3.2071 | 0.9396 | 3.41 | < 0.01 |
|  |  | Fire 150 | 4.4708 | 0.9698 | 4.61 | < 0.0001 |
|  |  |  |  |  |  |  |
|  | Fire 100 | Fire 120 | 0.7251 | 0.1678 | 4.32 | < 0.001 |
|  |  | Fire 150 | 1.9762 | 0.1683 | 11.74 | < 0.00001 |
|  |  |  |  |  |  |  |
|  | Fire 120 | Fire 150 | 1.2595 | 0.1668 | 7.55 | < 0.00001 |
|  |  |  |  |  |  |  |
|  |  |  |  |  |  |  |
| *U. parviflorus* | Control | Summer 5 | 1.3223 | 0.1772 | 7.46 | < 0.00001 |
|  |  | Summer 30 | 1.0610 | 0.2246 | 4.72 | < 0.0001 |
|  |  | Fire 80 | 2.1264 | 0.2154 | 9.87 | < 0.00001 |
|  |  | Fire 100 | 3.9165 | 0.4085 | 9.59 | < 0.00001 |
|  |  | Fire 120 | 4.2316 | 0.4281 | 9.88 | < 0.00001 |
|  |  | Fire 150 | 3.6527 | 0.3478 | 10.50 | < 0.00001 |
|  |  |  |  |  |  |  |
|  | Summer 5 | Summer 30 | -0.2618 | 0.1264 | -2.071 | ns |
|  |  | Fire 80 | 0.8106 | 0.2300 | 3.525 | < 0.01 |
|  |  | Fire 100 | 2.5983 | 0.2591 | 10.030 | < 0.00001 |
|  |  | Fire 120 | 2.9129 | 0.3314 | 8.791 | < 0.00001 |
|  |  | Fire 150 | 2.3401 | 0.2930 | 7.986 | < 0.00001 |
|  |  |  |  |  |  |  |
|  | Summer 30 | Fire 80 | 1.0745 | 0.2166 | 4.96 | < 0.00001 |
|  |  | Fire 100 | 2.8538 | 0.2835 | 10.07 | < 0.00001 |
|  |  | Fire 120 | 3.1671 | 0.4072 | 7.78 | < 0.00001 |
|  |  | Fire 150 | 2.5911 | 0.2899 | 8.94 | < 0.00001 |
|  |  |  |  |  |  |  |
|  | Fire 80 | Fire 100 | 1.7824 | 0.4490 | 3.97 | < 0.001 |
|  |  | Fire 120 | 2.0947 | 0.5314 | 3.94 | < 0.001 |
|  |  | Fire 150 | 1.5170 | 0.4170 | 3.64 | < 0.01 |
|  |  |  |  |  |  |  |
|  | Fire 100 | Fire 120 | 0.3146 | 0.2173 | 1.45 | ns |
|  |  | Fire 150 | -0.2643 | 0.2189 | -1.21 | ns |
|  |  |  |  |  |  |  |
|  | Fire 120 | Fire 150 | -0.5785 | 0.3287 | -1.76 | ns |
